# DCRM Multispecialty Practice Recommendations for the management of diabetes, cardiorenal, and metabolic diseases

**DOI:** 10.1016/j.jdiacomp.2021.108101

**Published:** 2021-12-07

**Authors:** Yehuda Handelsman, John E. Anderson, George L. Bakris, Christie M. Ballantyne, Joshua A. Beckman, Deepak L. Bhatt, Zachary T. Bloomgarden, Biykem Bozkurt, Matthew J. Budoff, Javed Butler, Samuel Dagogo-Jack, Ian H. de Boer, Ralph A. DeFronzo, Robert H. Eckel, Daniel Einhorn, Vivian A. Fonseca, Jennifer B. Green, George Grunberger, Chris Guerin, Silvio E. Inzucchi, Paul S. Jellinger, Mikhail N. Kosiborod, Pamela Kushner, Norman Lepor, Christian W. Mende, Erin D. Michos, Jorge Plutzky, Pam R. Taub, Guillermo E. Umpierrez, Muthiah Vaduganathan, Matthew R. Weir

**Affiliations:** a Metabolic Institute of America, Tarzana, CA, USA; b The Frist Clinic, Nashville, TN, USA; c University of Chicago Medicine, Chicago, IL, USA; d Baylor College of Medicine, Houston, TX, USA; e Vanderbilt University Medical Center, Nashville, TN, USA; f Brigham and Women’s Hospital, Harvard Medical School, Boston, MA, USA; g Mount Sinai School of Medicine, New York, NY, USA; h David Geffen School of Medicine, UCLA, Los Angeles, CA, USA; i University of Mississippi Medical Center, Jackson, MS, USA; j University of Tennessee Health Science Center, Memphis, TN, USA; k University of Washington, Seattle, WA, USA; l University of Texas Health Science Center, San Antonio, TX, USA; m University of Colorado Anschutz Medical Campus, Denver, CO, USA; n Scripps Whittier Institute for Diabetes, San Diego, CA, USA; o Tulane University Health Sciences Center, New Orleans, LA, USA; p Duke University Medical Center, Durham, NC, USA; q Grunberger Diabetes Institute, Bloomfield Hills, MI, USA, Wayne State University School of Medicine, Detroit, MI, USA, Oakland University William Beaumont School of Medicine, Rochester, MI, USA, Charles University, Prague, Czech Republic; r University of California San Diego School of Medicine, San Diego, CA, USA; s Yale School of Medicine, New Haven, CT, USA; t The Center for Diabetes & Endocrine Care, University of Miami Miller School of Medicine, Hollywood, FL, USA; u Saint Luke’s Mid America Heart Institute, University of Missouri–Kansas City, Kansas City, MO, USA; v University of California at Irvine, Irvine, CA, USA; w Johns Hopkins University School of Medicine, Baltimore, MD, USA; x Emory University, Atlanta, GA, USA; y University of Maryland School of Medicine, Baltimore, MD, USA

**Keywords:** Type 2 diabetes, Heart failure, Chronic kidney disease, Atherosclerotic cardiovascular disease, Clinical practice, Consensus recommendations

## Abstract

Type 2 diabetes (T2D), chronic kidney disease (CKD), atherosclerotic cardiovascular disease (ASCVD), and heart failure (HF)—along with their associated risk factors—have overlapping etiologies, and two or more of these conditions frequently occur in the same patient. Many recent cardiovascular outcome trials (CVOTs) have demonstrated the benefits of agents originally developed to control T2D, ASCVD, or CKD risk factors, and these agents have transcended their primary indications to confer benefits across a range of conditions. This evolution in CVOT evidence calls for practice recommendations that are not constrained by a single discipline to help clinicians manage patients with complex conditions involving diabetes, cardiorenal, and/or metabolic (DCRM) diseases. The ultimate goal for these recommendations is to be comprehensive yet succinct and easy to follow by the nonexpert—whether a specialist or a primary care clinician. To meet this need, we formed a volunteer task force comprising leading cardiologists, nephrologists, endocrinologists, and primary care physicians to develop the DCRM Practice Recommendations, a multispecialty consensus on the comprehensive management of the patient with complicated metabolic disease. The task force recommendations are based on strong evidence and incorporate practical guidance that is clinically relevant and simple to implement, with the aim of improving outcomes in patients with DCRM. The recommendations are presented as 18 separate graphics covering lifestyle therapy, patient self-management education, technology for DCRM management, prediabetes, cognitive dysfunction, vaccinations, clinical tests, lipids, hypertension, anticoagulation and antiplatelet therapy, antihyperglycemic therapy, hypoglycemia, nonalcoholic fatty liver disease (NAFLD) and nonalcoholic steatohepatitis (NASH), ASCVD, HF, CKD, and comorbid HF and CKD, as well as a graphical summary of medications used for DCRM.

## 1. Introduction

In the US, 88 million adults (35% of the population) have prediabetes, and 34 million (11%) have diabetes. Chronic kidney disease (CKD) currently affects 14% of the total US population, including up to 40% of those with diabetes.^1–3^ Both hyperglycemia and hypertension play an etiologic role in the development of CKD and heart failure (HF), and hypertension, hyperglycemia, and hyperlipidemia are all closely associated with obesity, metabolic syndrome, type 2 diabetes (T2D), CKD, atherosclerotic cardiovascular disease (ASCVD), HF and nonalcoholic fatty liver disease (NAFLD) and nonalcoholic steatohepatitis (NASH).^4–7^ Meanwhile, CVD remains the leading cause of death worldwide and in the US, where it affects nearly half of adults.^8–10^ The presence of CKD or diabetes doubles the risk of cardiovascular events, and when CKD and diabetes co-occur, risks are further increased.^10^

The confluence of these conditions calls for a holistic approach to treatment. Medical societies often develop practice recommendations to help manage patients with conditions specific to those disciplines. However, the results of recent cardiovascular outcome trials (CVOTs) transcend traditional applicability to a single medical specialty. Practice recommendations that are not constrained by a single discipline may help clinicians manage patients with complex conditions involving diabetes, cardiorenal, and/or metabolic (DCRM) diseases.

We therefore convened a volunteer task force comprising experts from multiple medical disciplines, including cardiologists, nephrologists, endocrinologists, and primary care physicians who are all recognized leaders in their respective fields, to develop recommendations across specialties in a holistic manner. The recommendations endorse a paradigm shift in management—preventing the next ASCVD/HF/CKD event independent of the level of risk factors while continuing to control traditional cardiovascular risks. The result is the DCRM Practice Recommendations, a multispecialty consensus on the comprehensive management of the patient with complicated metabolic disease based on strong evidence and incorporating practical guidance that is clinically relevant and simple to implement, with the aim of improving outcomes.

The recommendations consist of 18 separate slides organized into 3 sections (slide set downloadable at https://www.dcrmi.com/dcrm-practice-recommendations):

### Section I. General Health and Background Considerations

1. Elements of Lifestyle Therapy—Any Effort Is Worthwhile
2. Elements of Patient Self-Management Education: A Clinician’s Guide
3. Technology for Management of Diabetes, Cardiorenal, and Metabolic Diseases
4. Prediabetes: A Continuum of Cardio-Renal-Metabolic Risk
5. Preventing and Managing Cognitive Dysfunction
6. Vaccinations for People with Diabetes, Cardiorenal, or Metabolic Diseases
7. Clinical Tests for Diabetes, Cardiorenal, and Metabolic Diseases

### Section II. Traditional Cardiovascular Risk Management

- Management of Lipids in Diabetes, Cardiorenal, and Metabolic Diseases
- Management of Hypertension in Diabetes, Cardiorenal, and Metabolic Diseases
- Principles of Anticoagulation and Antiplatelet Therapy
- Antihyperglycemic Therapy
- Management of Hypoglycemia

### Section III. Contemporary Prevention of Comorbidities and Mortality

- Management of NAFLD and NASH
- Management of ASCVD
- Prevention and Management of Heart Failure
- CKD Diagnosis and Treatment
- Management of Comorbid Heart Failure and CKD
- Summary of Medications for Diabetes, Cardiorenal, and Metabolic Diseases

## 2. DCRM Multispecialty Practice Recommendations

### 2.1. Section I. General Health and Background Considerations

#### 2.1.1. *Elements of Lifestyle Therapy*—*Any Effort Is Worthwhile*

Optimizing lifestyle can improve quality and quantity of life, even in complex patients.

Good mental health is the cornerstone of a healthy lifestyle. Mood disturbances, substance abuse, prior personal traumas, and psychosocial limitations should be addressed and the patient referred as necessary. Encourage positive practices such as mindfulness and engagement with social activities.

Nutrition is of paramount importance, with caloric restriction to enable 5% to 10% weight loss in persons with overweight or obesity.^11^ Encourage fruits, vegetables, whole grains, and legumes and discourage unhealthy foods.^12,13^ Short-term continuous glucose monitoring (CGM) may help understand the impact of food and exercise on glucose.^14,15^ Many popular diets work—and recommendations may be individualized based on patient preference—but it is important to emphasize that healthy eating is for the long term. Recidivism is common.

For most people, at least 150 min per week of moderate intensity aerobic plus resistance activity is recommended. Encourage apps and devices to motivate and monitor activity.

Sleep duration (at least 7 h) and quality are often under appreciated. Sleep deprivation worsens insulin resistance, hypertension, hyperglycemia, and dyslipidemia and increases inflammatory cytokines. Adequate sleep on a nightly basis may decrease these risks.^16^ People with obesity and diabetes have an increased prevalence of sleep apnea, and appropriate treatment can help improve a patient’s quality of life and comorbidities.^17^ Sleeping pills are generally unhelpful and can have serious side effects.

Smoking cessation is the single most important step, and a clinician’s encouragement is cited as a frequent motivator to quit smoking. Excess alcohol intake can contribute to weight gain, hypertension, cardiomyopathy, and atrial fibrillation, as well as peripheral neuropathy, fatty liver, and dementia—all issues in this population. Patients should consume no more than 1–2 daily drinks per day (women, ≤1 drink per day; men, ≤2 drinks per day of 12 oz of beer, 5 oz of wine, or 1.5 oz of distilled spirits).^18^

#### 2.1.2. Elements of Patient Self-Management Education: a Clinician’s Guide

Self-management education is for all patients with cardiorenal and/or metabolic diseases, not just those with diabetes. The goal of patient education is to improve adherence to therapeutic interventions—both lifestyle *and* medications—by increasing patients’ understanding of their medical conditions and how to reduce associated risks.

Patient education carries many challenges, including high costs in time and resources and the need to tailor communication to each individual’s health literacy and socioeconomic circumstances. Patients with diabetes should be referred to diabetes care and education specialists (CDCES; formerly known as certified diabetes educators [CDEs]), if available, for disease-specific training. Telehealth may allow clinicians to provide education to multiple patients in a group setting.

Clinicians should explain—in plain language—all the different examinations and tests patients might undergo. A goal for all patients is that they “know their numbers” and have a basic understanding of what each means for their health (Table 1).

Patients require ongoing education and reinforcement on the importance of a healthy lifestyle. In addition, all medications should be explained so that patients fully understand what the medication does, when they should take it, what side effects may occur, and what to do if a side effect is serious or extremely bothersome. Finally, offering patients support and information on navigating the healthcare system can help address healthcare disparities and improve health outcomes.

Medication reconciliation helps drive discussions about adherence to treatment. Shared decision making and motivational interviewing techniques can help ensure patient education is tailored to individual patient needs.^19,20^

#### 2.1.3. Technology for Management of Diabetes, Cardiorenal, and Metabolic Diseases

Health-aid technology has proliferated in recent years alongside innovations in smart phones and other hand-held information aids. Patients should be encouraged to use validated apps (for smart phones, tablets, and/or computers) to help track components of their lifestyle therapy regimens. The use of such apps has been shown to improve activity levels, dietary quality, and weight and blood pressure (BP) control.^21,22^ Likewise, the use of wearable fitness trackers leads to increased frequency and duration of physical activity.^23^

Ambulatory and home BP monitoring can help distinguish between normotension and masked hypertension and between white coat and sustained hypertension. Out of office BP readings more accurately predict morbidity and mortality than in-office readings.^24,25^

In addition to technologies used to track general health measures, various devices for glucose monitoring and insulin delivery can help improve glycemic control in patients with diabetes. The evidence supporting these devices has been thoroughly reviewed in recent diabetes management guidelines.^26,27^

CGM is increasingly becoming a mainstay of diabetes management, allowing patients and clinicians to track time in range and hyper- and hypoglycemic excursions to more closely tailor antihyperglycemic regimens. CGM is also an important tool for patients at risk from severe hypoglycemia due to nondiabetic causes (e.g., refractory insulinoma, nesidioblastosis, postbariatric hypoglycemia, etc.). These devices may be owned and used continuously by patients (*personal* CGM) or owned by the clinical practice and used intermittently to identify glycemic patterns that are undetectable with A1C monitoring (*professional* or *diagnostic* CGM). Models that include hyper- and hypoglycemic alarms and remote monitoring provide important safeguards for patients. CGM data may help patients understand the impact of lifestyle choices on their blood glucose.

For patients without access to CGM who take insulin, sulfonylureas, or glinides, structured self-monitoring of blood glucose (SMBG) with traditional fingerstick blood glucose monitors should be used. *Structured* refers to SMBG regimens comprising a predefined testing schedule and interpretation of the data with the patient to inform clinical decision making.^28^

Continuous subcutaneous insulin infusion (CSII) via insulin pumps, with appropriate training, may be preferred for many patients treated with intensive insulin regimens (basal insulin plus prandial insulin for ≥2 meals per day)—primarily patients with type 1 diabetes (T1D) and some with T2D—because of documented improvements in glycemic control and diabetes outcomes relative to those using multiple daily injections (MDI).^26,27^ Models that integrate a CGM device and insulin pumps including a mechanism that stops insulin infusion during hypoglycemic episodes and modulates infusions to achieve predetermined preprandial glucose levels (i.e., hybrid close loop systems) may be preferred for patient safety.


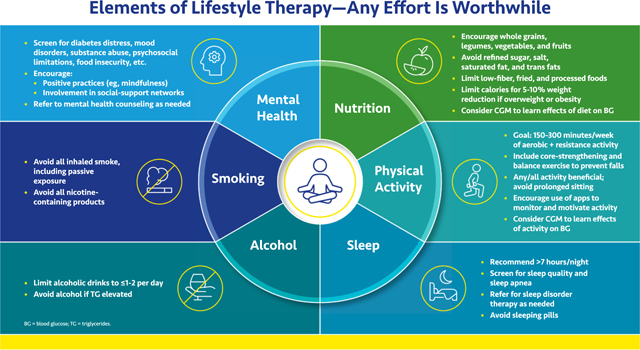



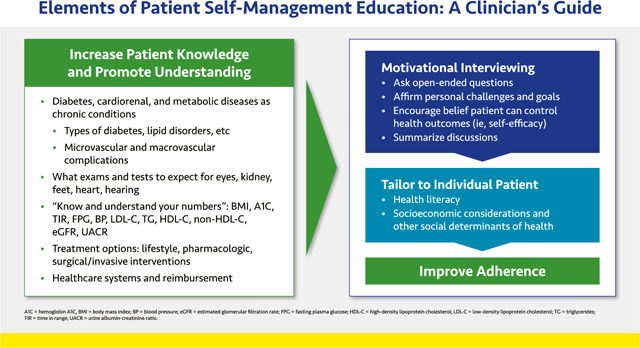



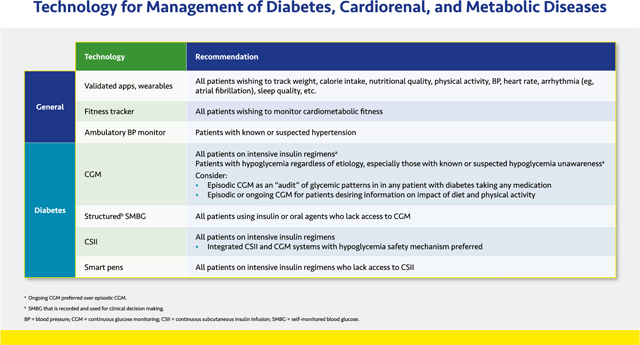



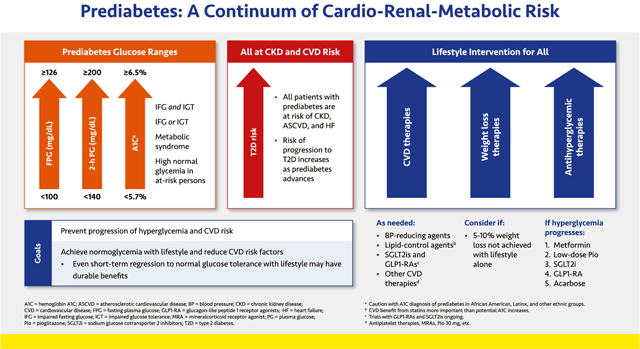


“Smart” insulin pens, which capture data on insulin dosing and incorporate this information with glucose excursion data from glucose monitors and connect wirelessly to diabetes management software, are suitable for people on insulin injections.

#### 2.1.4. Prediabetes: a Continuum of Cardio-Renal-Metabolic Risk

Prediabetes is a continuum of metabolic abnormalities that extend from the metabolic syndrome with high normal glucose through increasingly severe glucose abnormalities, including impaired fasting glucose (IFG) and/or impaired glucose tolerance (IGT) to just below the diagnostic thresholds for T2D. Metabolic syndrome is defined according to the National Cholesterol Education Program Third Adult Treatment Panel (NCEP ATP III; i.e., at least 3 of the following: elevated fasting glucose, triglycerides, or BP; low high density lipoprotein-cholesterol [HDL–C], and abdominal obesity).^29^ Patients with prediabetes have increased risks of ASCVD, HF, and CKD.^5,30,31^ It is therefore essential to optimally control BP, lipids, and other CVD risk factors (see below).

Progression from normoglycemia to overt T2D results from a progressive beta-cell defect.^32^ Among patients with prediabetes, the incidence of T2D is up to 8% to 11% per year, and roughly half of patients at risk for T2D never develop it. Evidence suggests that intervening early, when dysglycemia is less severe, may prevent progression to T2D or even foster reversion to normoglycemia.^33,34^ Prediabetes therapy is therefore focused on identifying and educating the person at risk with an emphasis on lifestyle modification, weight management, control of CVD risk factors, and efforts to revert to normoglycemia.

Sustained weight loss increases the likelihood of achieving normoglycemia.^34^ If a weight loss of at least 5% to 10% cannot be achieved with lifestyle therapy alone, pharmacological and surgical intervention may be considered depending on the patient’s underlying comorbidities as well as body mass index (BMI).^11,35^ Although lifestyle therapy is the most effective and durable approach to delaying or preventing T2D onset in patients with prediabetes, pharmacological therapy with metformin, acarbose, or pioglitazone has been shown to decrease the risk of overt T2D.^36–39^ Accumulating data suggest that glucagon-like peptide 1 receptor agonists (GLP1-RAs), and to a lesser extent sodium glucose cotransporter 2 (SGLT2) inhibitors, may be quite potent in delaying or preventing progression to T2D, probably in part through their weight loss effects.^40,41^ Although the US Food and Drug Administration (FDA) has not approved any of these medications to prevent T2D, based on clinical research results, the American Diabetes Association (ADA), American Association of Clinical Endocrinologists (AACE), and the International Diabetes Federation (IDF) recommend their use in high-risk patients.

#### 2.1.5. Preventing and Managing Cognitive Dysfunction

Cognitive dysfunction refers to various forms of major neurocognitive disorder (previously known as dementia), characterized by a decrease from previous level of performance in one or more cognitive domains (learning and memory, language, executive function, complex attention, perceptual-motor, social cognition).^42^ All forms of dementia including the most common forms (Alzheimer’s disease [AD] and vascular dementia) may be complications of diabetes or cardiovascular disease.

Age is the primary risk factor for cognitive dysfunction.^43^ Vascular dementia may have a sudden onset and progress in a stepwise fashion, whereas AD usually has a more gradual onset and progression; both frequently co-occur in the same patient.^44^ Diabetes increases the risk of AD by 56% and vascular dementia by 127%, and atrial fibrillation and HF more than double the risk of dementia.^45,46^ Recurrent, severe hypoglycemia and hearing loss also increase cognitive impairment risk.^47,48^

Screening (Mini-Mental State Examination [MMSE] and the Clock Drawing Test) and diagnosis of cognitive dysfunction can be done in the primary care office, with referral as needed. Patient history is critical and structural imaging is useful to identify vascular dementia–related brain injuries.^44^

All components of DCRM and other clinical comorbidities should be addressed, as needed. Clinicians should monitor behavioral and emotional function as patients age and refer for neuropsychiatric evaluation as needed.

The etiology of AD is not well understood, and pharmacological agents have not yet shown conclusive benefits. Aducanumab, an anti-amyloid antibody therapy, reduces beta-amyloid plaque with some symptomatic benefit. However, full approval is contingent on the results of an ongoing phase 4 randomised trial.^49^ Several other anti-amyloid agents remain under investigation.

Most treatment options approved for AD do not address the underlying etiology but rather stall symptomatic decline. The cholinesterase inhibitors modestly improve cognition, activities, and behavior, but the long term benefits of these agents are unclear. No agents are currently approved to treat cognitive deficits caused by vascular dementia. All patients should be encouraged to consider advanced care planning.

#### 2.1.6. Vaccinations for People with Diabetes, Cardiorenal, or Metabolic Diseases

The Centers for Disease Control and Prevention (CDC) has designated individuals with diabetes, cardiovascular, kidney, and other chronic metabolic diseases as priority groups for vaccination because they are at high risk of complications from infections—especially from the influenza virus and severe acute respiratory syndrome coronavirus 2 (SARS-CoV-2), the cause of coronavirus disease 2019 (COVID-19).^50–54^ These respiratory infections may worsen or even cause long-term cardiovascular and renal complications, and vaccination can reduce these outcomes.^54–58^


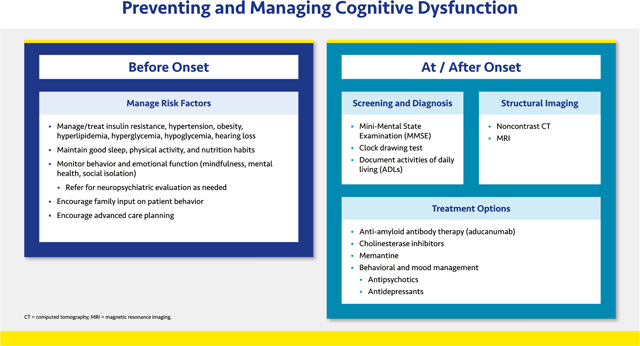



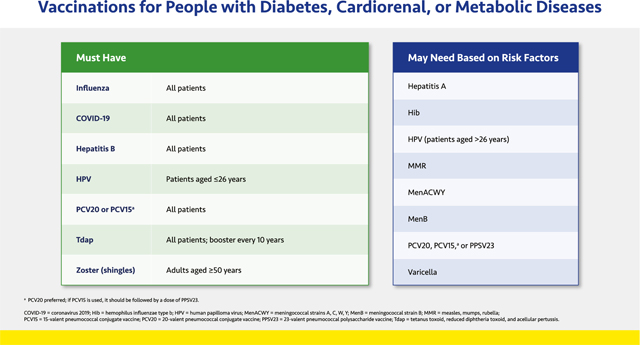



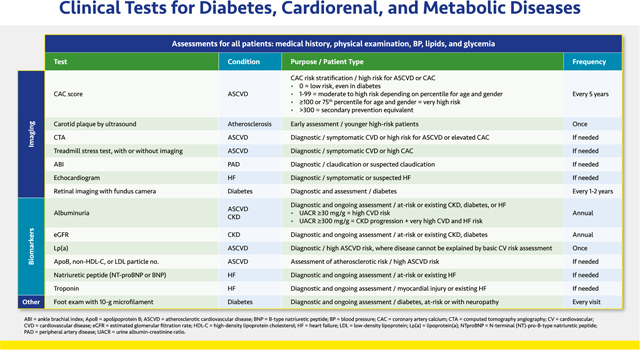


The vaccinations listed in the slide were compiled based on CDC recommendations and apply to all adults with diabetes, CVD, or CKD.^52,53^ If the patient does not know their vaccination status, it is advisable to administer the vaccine in question, because the benefits of protection far outweigh the negligible risks of an extra dose.

#### 2.1.7. Clinical Tests for Diabetes, Cardiorenal, and Metabolic Diseases

The guidance lists the most commonly performed clinical assessments beyond the standard tests for BP, lipids, and glucose; others may be necessary for the individual patient. Clinicians should explain the purpose of all clinical examinations to patients (see Section 2.1.2. Patient Education).

The coronary artery calcium (CAC) score uses computed tomography (CT) to stratify ASCVD risk based on the amount of calcium in arterial walls, which is a surrogate marker for the total atherosclerotic plaque burden. The CAC score may be a useful tool in low to intermediate risk patients. The CAC score may be repeated every ~5 years people with very low CAC.^59^ Computed tomography angiography (CTA) or a treadmill stress test with or without imaging may also be used to help diagnose ASCVD.^60,61^ Additional imaging tests used primarily for diagnosis include ultrasound of carotid plaque and the ankle brachial index (ABI).^62,63^ Echocardiography may be used in patients with symptomatic or suspected HF.^64^ The Task Force does not recommend measurement of carotid intima media thickness (IMT) in clinical practice or use of the treadmill stress test, 6-min walking test, or ABI for routine screening.

Annual screening for diabetic retinopathy should be done by an ophthalmologist or by retinal imaging. Retinal images should be interpreted by trained eye care providers and if positive should be referred to an ophthalmologist immediately.^30^

Albuminuria and estimated glomerular filtration rate (eGFR) are used to diagnose and monitor CKD in patients with or at risk of CKD as well as those with diabetes (see Section 2.3.4. CKD). Experts no longer recommend classifying urinary albumin levels as *microalbuminuria* and *macroalbuminuria*. Any level of persistent albuminuria (i.e., urine albumin-creatinine ratio [UACR] ≥30 mg/g for >3 months) suggests at least a moderate risk of CKD progression as well as an increased risk of ASCVD. Patients with UACR ≥300 mg/g are at high risk of CKD progression, as are patients with eGFR ≤44 mL/min/1.73 m^2^.^4,7^

Lipid parameters are important in predicting cardiovascular risk (see Section 2.2.1. Lipids). Lipid panel measurement provides low-density lipoprotein cholesterol (LDL-C) and non-HDL-C, which is important in patients with hypertriglyceridemia. Other important measures are apolipoprotein B (apoB) elevations and LDL particle number. Elevated lipoprotein (a) [Lp(a)] suggests enhanced ASCVD risk in those with a family history of premature ASCVD or personal history of ASCVD not explained by major risk factors. The Task Force does not recommend Lp(a) for routine screening purposes.^59^

In those with or at risk of HF (see Section 2.3.3. Heart Failure), both natriuretic peptides (N-terminal [NT]-pro-B-type natriuretic peptide [NT-proBNP] or BNP) and high-sensitivity troponin T or I (hs-TnT or hs-TnI) are useful biomarkers of the presence and severity of HF.^64,65^ Natriuretic peptides may be particularly useful in the differential diagnosis of HF when the cause of dyspnea is unclear.^65^ Elevated troponin indicates myocyte injury or necrosis in patients with myocardial injury or diagnosed HF.^64,65^

Finally, patients with diabetes and evidence of sensory loss or history of ulceration or amputation should have their bare feet examined at every office visit. In other patients, a comprehensive foot examination should be performed annually.^30^

### 2.2. Section II. Traditional Cardiovascular Risk Management

#### 2.2.1. Management of Lipids in Diabetes, Cardiorenal, and Metabolic Diseases

Lipid management should be based on patients’ comorbidities, which, along with their baseline lipid levels, informs their level of risk. A wealth of data from outcomes trials with statins, ezetimibe, and pro-protein convertase subtilisin/kexin type 9 (PCSK9) inhibitors have shown that for patients with elevated low-density lipoprotein cholesterol (LDL-C), there appears to be no threshold of benefit for LDL-C level—so far, lower is always better.^66–69^ It is recommended to reduce LDL-C levels by at least 50% from baseline or reach the patient’s risk-based goal, whichever is lower. Achieving these levels often requires combination therapy.

Risk-based goals shown in the slide are based on guideline recommendations from the American College of Cardiology (ACC) and American Heart Association (AHA), the AACE, and the European Society of Cardiology (ESC) and European Atherosclerotic Society (EAS) (Table S1).^6,59,70,71^ The *extreme plus* goal (<40 mg/dL [<104 mmol/L]) is for patients who have an extreme risk and continue to have cardiovascular events despite LDL-C <55 mg/dL (<1.42 mmol/L).^71^ Ten-year risk, which refers to the risk of a hard ASCVD event (myocardial infarction, coronary heart disease death, non-fatal or fatal stroke) within 10 years, can be calculated using a validated risk calculator chosen based on the patient’s characteristics (Table S2).^72–78^ The Multiethnic Study of Atherosclerosis (MESA; https://www.mesa-nhlbi.org/CAC-Tools.aspx ) calculator is preferred because it incorporates the CAC score.^72^


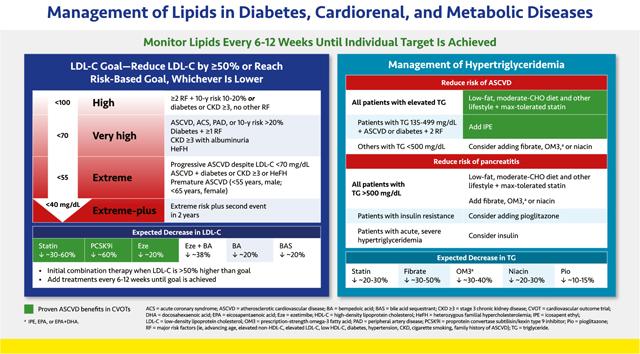


Major risk factors include those that increase atherosclerotic risk, including advancing age, elevated LDL-C or non-HDL-C, low HDL-C, diabetes, hypertension, CKD, cigarette smoking, and family history of ASCVD.

Drug classes that lower LDL-C and are highlighted in green have proven benefits in CVOTs.^67–69^ All patients at elevated ASCVD risk should receive a statin at the maximally tolerated dose unless there is a contraindication. If the baseline LDL-C is more than 50% above the goal, initial combination therapy with a statin plus ezetimibe, bempedoic acid, or a PCSK9 inhibitor should be instituted. The choice of the second or third agent to add to statin therapy is based on how much LDL-C lowering is required to reach the LDL-C goal. Whether treatment begins with a statin alone or in combination with another agent, therapy should be intensified every 6–12 weeks until the LDL-C goal is achieved.

Patients with homozygous familial hypercholesterolemia should be referred to a lipid specialist. In these patients, adding the monoclonal antibody evinacumab, an inhibitor of angiopoietin-like 3 (ANGPTL3), to other lipid-lowering therapy reduces LDL-C levels by a further ~50%.^79^ The microsomal triglyceride transfer protein (MTP) inhibitor lomitapide may also be considered, although some potential for hepatotoxicity exists.

The generally accepted therapeutic goal for triglycerides of <150 mg/dL (<1.7 mmol/L) was defined in 2001 by the NCEP ATP III,^80^ although studies suggest that optimal triglyceride levels may be lower.^81^ Elevated triglycerides (>150 but <500 mg/dL [>1.7 but <5.7 mmol/L]) should be managed with maximum tolerated statin therapy and a heart healthy, moderate-carbohydrate diet with restricted simple sugar and alcohol intake in addition to other lifestyle approaches (see Section 2.1.1. Lifestyle Therapy). Based on evidence from the Reduction of Cardiovascular Events with Icosapent Ethyl—Intervention Trial (REDUCE-IT) trial, adding icosapent ethel (IPE), a highly purified, non-oxidized formulation of the omega-3 fatty acid eicosapentaenoic acid (EPA), to statin therapy further reduces the risk of ASCVD events in patients with triglycerides between 135 and 500 mg/dL (1.5–5.7 mmol/L) who have ASCVD or diabetes plus two major ASCVD risk factors.

With the exception of the Helsinki Heart Study and the pre-statin era Veterans Affairs High-Density Lipoprotein Cholesterol Intervention Trial (VA-HIT) with gemfibrozil, CVOTs involving triglyceride-reducing agents, including fenofibrate, omega-3 fatty acids other than IPE, and niacin, have not demonstrated reductions in ASCVD.^6^ However, subgroup analyses from some trials showed a trend toward benefit in patients with triglycerides ≥200 mg/dL (≥2.3 mmol/L) and HDL-C ≤ 40 mg/dL (≤1.0 mmol/L) who received fibrates.^82–85^

Patients with severely elevated triglycerides (>500 mg/dL [>5.7 mmol/L]) are at risk of pancreatitis. These patients should be prescribed dietary restriction and other lifestyle therapeutic approaches along with a fibrate, omega-3 fatty acid, or niacin to reduce pancreatitis risk.

Fibrates, which reduce triglycerides by up to 50%, are considered the most potent triglyceride-lowering agents.^66,86^ Because of increased risk of myopathy, fibrates should be used with caution in combination with certain statins. Although fenofibrate is typically safer with most statins, this agent should also be used cautiously with some statins (e.g., simvastatin). Prescription-grade omega-3 fatty acids, including IPE, other EPA-only formulations, or EPA plus docosahexaenoic acid (DHA), reduce triglycerides by up to 40%.^66,87^ Niacin, or nicotinic acid, reduces triglycerides by up to 30%. Because it is associated with glucose elevations, it should be used cautiously if at all in people with diabetes or prediabetes.^66^ Depending on the severity of triglyceride elevations, more than one of these agents may be needed. Pioglitazone may also be useful in patients with insulin resistance. Those with acute, severe hypertriglyceridemia and hyperglycemia may benefit from insulin infusion.^5,66^

#### 2.2.2. Management of Hypertension in Diabetes, Cardiorenal, and Metabolic Diseases

Current guidelines recommend a BP <130/80 mmHg to slow CKD progression.^88,89^ Systolic BP (SBP) levels ≥140 mmHg have been associated with faster declines in kidney function. In addition, those with more than 1 g/day of albuminuria regardless of CKD etiology and/or a history of stroke benefit from SBP levels <120 mmHg.^90,91^ On average, ~3 BP medications are needed to achieve a SBP <130 mmHg. For any patient with albuminuria and hypertension, the BP-lowering regimen should include a renin-angiotensin system (RAS) inhibitor at maximal dose, a calcium channel blocker (CCB), and a thiazide-type diuretic such as chlorthalidone or indapamide.^88,89,92,93^ Chlorthalidone or indapamide are preferred over hydrochlorothiazide (HCTZ) because they have a longer half-life and reduce mortality compared to HCTZ.^92,94^


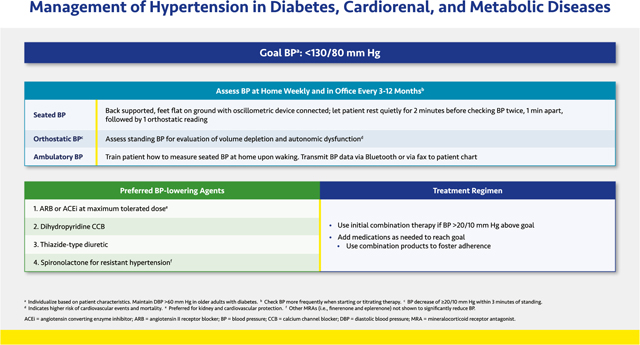


When treating people with SBP well above 150–160 mmHg, rapidly controlling BP will lead to an increase of up to 30% in serum creatinine, regardless of RAS inhibition. This effect is expected and temporary; it is *not* a sign of acute kidney injury. If therapy is continued, the creatinine elevation will resolve over the span of a week or so. Moreover, increases in creatinine in response to BP treatment should not be concerning unless hyperkalemia develops or creatinine continues to rise above 30%. The most common cause of this problem is pre-existing volume depletion, and the patient should be hydrated.^95^

Out-of-office assessment of BP is very important because white coat and especially masked hypertension (i.e., higher BP elevations outside than inside the clinic) are commonly missed and will affect BP treatment. These conditions must be diagnosed with home BP monitoring and the measurements compared with office readings. The cardiovascular mortality risk may be increased in patients with white coat or masked hypertension.^24,96^ For many of these patients, pharmacologic and/or behavioral therapies for anxiety and other stress-related disorders may be needed in addition to antihypertensive medication.

#### 2.2.3. Principles of Anticoagulation and Antiplatelet Therapy

Determination of the optimal antithrombotic therapy is complex. For patients without a history of ASCVD or other risk factors, the use of aspirin is not generally recommended. However, A Study of Cardiovascular Events in Diabetes (ASCEND) demonstrated a modest reduction in ischemic events for patients with diabetes but without ASCVD, although an increased risk of bleeding was similar in magnitude to the benefit.^97^ The Task Force believes prescribing aspirin for patients with two or more cardiovascular risk factors (i.e., advanced age, elevated non-HDL-C, elevated LDL-C, low HDL-C, diabetes, hypertension, CKD, cigarette smoking, family history of ASCVD, elevated CAC score > 100) may benefit those who are not at increased risk of bleeding.

The use of antithrombotic therapy in patients with atherosclerosis has been studied in various clinical trials. In the setting of an acute coronary syndrome (ACS), dual antiplatelet therapy (DAPT) consisting of aspirin with a P2Y12 inhibitor for at least 12 months has been found to reduce the incidence of recurrent myocardial infarction (MI) and death.^98–100^ Both prasugrel and ticagrelor have demonstrated superiority compared with clopidogrel.^99,100^ In ACS treated with percutaneous coronary intervention (PCI), ticagrelor is also superior to clopidogrel.^99^ Prasugrel should be avoided in patients with history of transient ischemic attack (TIA) or stroke. As many as 5% of patients prescribed ticagrelor stop the medication because of shortness of breath.^101^

In patients who have not had any bleeding but remain at high ischemic risk, durations of DAPT longer than 12 months are recommended. Bleeding risk should be periodically reassessed.

Patients with stable CAD undergoing PCI should be treated with DAPT for at least 6 months if there are no bleeding complications. Thereafter, continued DAPT is reasonable if there has been no bleeding and they remain at high ischemic risk, although de-escalation to either clopidogrel or aspirin monotherapy may be considered if the bleeding risk is not low. In patients with a more remote history of PCI who are not receiving DAPT but are still at high ischemic risk and at low bleeding risk, either aspirin plus ticagrelor or dual pathway inhibition (DPI), which consists of rivaroxaban 2.5 mg twice daily plus aspirin 75–100 mg, should be considered.^102–105^ For high-risk patients with stable CAD and no prior PCI, either DPI or clopidogrel alone is acceptable; either clopidogrel or low-dose aspirin may be used for those at moderate risk. Finally for patients with PAD, DPI is recommended after revascularization, and DPI or clopidogrel alone is acceptable in the setting of PAD without revascularization.^106,107^

#### 2.2.4. Antihyperglycemic Therapy

The emergence of antihyperglycemic therapies with nonglycemic benefits permits a two-pronged approach to managing T2D. Concomitant with lifestyle therapy, patients with T2D with established or at high risk for ASCVD, CKD and/or HF should be prescribed antihyperglycemic agents proven to decrease the corresponding risks independent of their effects on glucose—namely, SGLT2 inhibitors and long-acting (LA) GLP1-RAs. The consensus includes pioglitazone as another agent with cardiovascular benefits, especially in stroke patients, with appropriate cautions about HF. Once the cardiovascular, CKD, or HF conditions have been addressed, antihyperglycemic regimens aimed at glucose reduction should be implemented to meet glycemic goals for the individual patient.


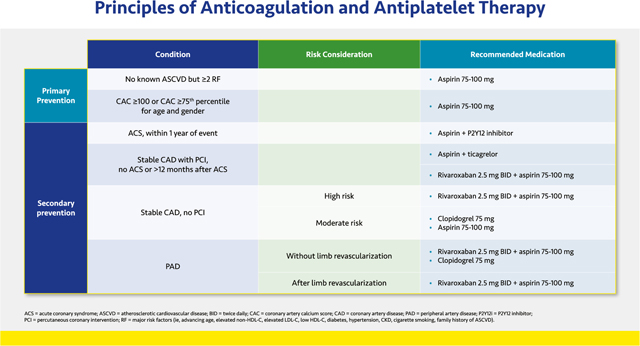



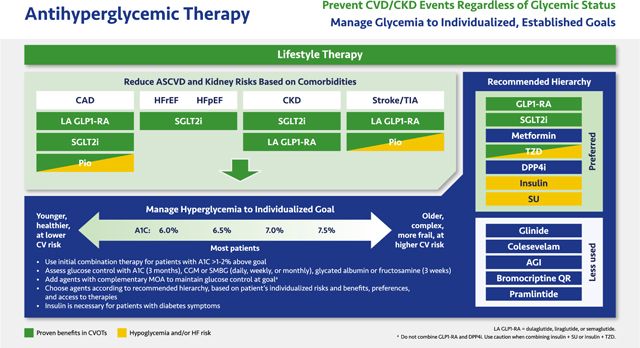


In the pale green box, classes with proven benefits are listed beneath each comorbidity according to the strength of CVOT evidence of benefit. CVOTs support cardiovascular safety but not benefits of the GLP1-RAs lixisenatide, twice daily or once weekly exenatide, and oral semaglutide, so these members of the class are recommended for glycemic control but not ASCVD or renal risk reduction.^108–110^ In contrast, the LA GLP1-RAs dulaglutide, liraglutide, and injectable semaglutide reduce the risk of major adverse cardiovascular events (MACE), including cardiovascular deaths, nonfatal MI, and especially nonfatal strokes, probably through mechanisms that affect atherosclerosis and improve myocardial contractility and endothelial function.^111–113^ Therefore, these agents are listed first under CAD and cerebrovascular disease (stroke/TIA). The majority of the GLP1-RA CVOTs involved patients with established CVD, while dulaglutide also included patients with multiple risks for CVD.^111–113^ Kidney benefits with LA GLP1-RAs were statistically significant in clinical trials; however, further dedicated studies are warranted.^112–114^

In outcome studies, all SGLT2 inhibitors reduced the risk of HF hospitalizations and improved kidney function.^115–122^ Other cardiovascular endpoints were improved with canagliflozin, which prevented MACE; empagliflozin, which reduced cardiovascular death in patients with established CVD; dapagliflozin, which prevented HF hospitalization in patients without prior ASCVD; and the dual SGLT1/SGLT2 inhibitor sotagliflozin (which is not approved in the US), which reduced MACE and HF.^116–120^ Recent studies with dapagliflozin, empagliflozin, and sotagliflozin have also demonstrated that these agents improve outcomes in patients with HF with reduced and with preserved ejection fraction (HFrEF and HFpEF, respectively), including patients who do not have T2D (dapagliflozin, empagliflozin).^40,119,123–125^ Canagliflozin and dapagliflozin improved CKD and reduced ASCVD and HF events in patients with moderate to severe CKD, and dapagliflozin showed similar effects in patients without diabetes.^121,126^

The thiazolidinedione (TZD) pioglitazone is included under CAD and stroke/TIA by expert consensus. This agent reduced the risk of the composite of all-cause mortality, non-fatal MI, or stroke in patients with T2D based on the secondary outcome of the Prospective Pioglitazone Clinical Trial in Macrovascular Events (PROActive).^127^ In the Insulin Resistance Intervention after Stroke (IRIS) trial, pioglitazone significantly reduced stroke and MI risk in a nondiabetic stroke population with insulin resistance, many of whom had prediabetes.^128^

Glycemic control efforts should be tailored to individualized goals for A1C and other glucose measures. As elaborated in recommendations from AACE and ADA, an A1C goal between 6.5% and 7.0% is appropriate for most patients. Younger, healthier patients at lower cardiovascular risk may benefit from A1C goals closer to normal (<6.0%), whereas higher A1C goals (~7.5% or higher) may be appropriate for older patients with more complex disease complicated by multiple comorbidities. In general, glycemic control regimens should aim to achieve and maintain the lowest A1C possible without hypoglycemia or other unacceptable side effects.^5,129,130^

Combination therapy should be instituted for patients whose A1C is >1% to 2% above their individualized goal, even in newly diagnosed T2D. Combination therapy should involve agents with complementary mechanisms of action; do not combine the incretin classes (GLP1-RAs and dipeptidyl peptidase 4 [DPP4] inhibitors) with each other or combine sulfonylureas with glinides. The Task Force recommends choosing agents according to the top-down hierarchy shown, although the patient’s characteristics, preferences, and access to therapies should be considered. In the list of *Preferred* classes, the GLP1-RAs and SGLT2 inhibitors are positioned above metformin because not only do they have cardiovascular benefits but also because they reduce weight and BP in addition to glucose. Many patients will need combination therapy with one or both of these classes as well as metformin, although the sequence should be individualized. Insulin is associated with weight gain and the risk of hypoglycemia. However, insulin should not be withheld from patients who cannot meet their glucose goals using other agents, and insulin should be used in any patient exhibiting the symptoms of uncontrolled diabetes (i.e., polyuria, polydipsia, or polyphagia). Finally, sulfonylureas carry an increased risk of hypoglycemia and weight gain with little benefit beyond rapid and relatively potent, albeit short-term, glycemic reductions. The *Less used* classes—glinides, colesevelam, alpha glucosidase inhibitors (AGIs), bromocriptine quick release (QR), and pramlintide—may be appropriate for individual patients in specific circumstances.^5,129,130^

Glycemic control should be evaluated on an ongoing basis. A1C reflects the average glucose level over 3 months and is the gold standard glycemic measure, although it has significant limitations. Other glycemic indices, such as time in range (TIR) data from patients’ CGM or SMBG devices, glycated albumin, or fructosamine, provide valuable information.^5,129–132^


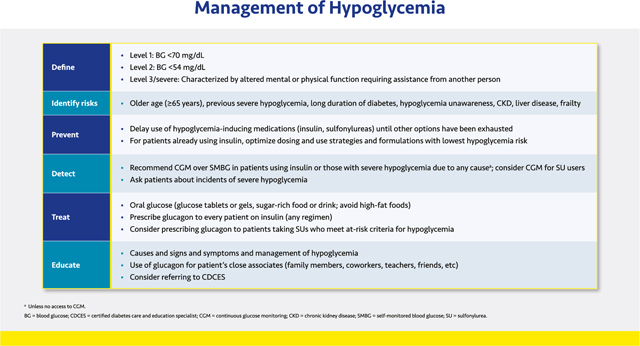


#### 2.2.5. Management of Hypoglycemia

Prevention and treatment of hypoglycemia is essential to cardiometabolic management in patients with diabetes and other conditions such as refractory insulinoma, nesidioblastosis, or postbariatric hypoglycemia. Mild, acute hypoglycemia can impair thinking or result in accidents or falls, while the long-term risk of sudden death, autonomic neuropathy, silent myocardial ischemia, cardiac arrhythmia, cognitive impairment, cerebrovascular disorders, and other adverse outcomes increases dramatically with recurrent and/or severe hypoglycemia.^133,134^ Fear of hypoglycemia complications poses a significant barrier to glycemic control in T2D and contributes to under-treatment of the disease.^135,136^ Approximately 25% of patients with diabetes have an A1C >8.0%, even though the risks of hypoglycemia do not diminish, and may even increase, at higher A1C levels.^137–139^

Hypoglycemia is categorized as level 1 (blood glucose <70 mg/dL [<3.9 mmol/L]), level 2 (blood glucose <54 mg/dL [<3.0 mmol/L]), and level 3 (severe hypoglycemia), or an event that involves an altered mental state requiring assistance from another person.^130,132^

Preventing hypoglycemia begins with the choice of antihyperglycemic therapy. Classes that do not induce hypoglycemia should be used in preference to insulin, sulfonylureas, and glinides, unless individualized glycemic targets cannot be met otherwise (see Section 2.2.4. Antihyperglycemic Therapy and Section 2.3.6. Medications Summary). For patients who require insulin, dosing should be optimized with insulin analogs (rather than human insulins) to minimize hypoglycemia risk.^5,129^

Utilizing CGM systems that alert patients of downward trends in blood glucose may prevent hypoglycemia.^132^ Patients without access to CGM but who use insulin or oral hypoglycemic agents should test their blood glucose frequently using a structured SMBG regimen.

Patients experiencing hypoglycemia should treat it by consuming 15 g of carbohydrate in the form of glucose tablets or gels or drink but avoid high-fat foods such as ice cream, which may slow glucose absorption. If hypoglycemia is not resolved within 15 min (i.e., glucose remains <70 mg/dL [<3.9 mmol/L]), a 15-gram carbohydrate load should be repeated. Every patient taking insulin—even basal-only regimens—should be prescribed glucagon to treat severe hypoglycemia, and the patient’s family members and other close associates should be trained in how to administer it. Glucagon may also be considered for patients taking sulfonylureas who meet criteria for high hypoglycemia risk. Lack of training for caregivers in glucagon administration or fears of causing additional harm often lead to unnecessarily prolonged episodes of severe hypoglycemia.^140^ Newer glucagon formulations, including nasal glucagon, single-dose auto-injector glucagon, or dasiglucagon pens, are easier to use than traditional glucagon kits, which can facilitate training.^141^

All patients and their family members and caregivers should be given education in the causes of hypoglycemia and how to prevent, detect, and treat it.

### 2.3. Section III. Contemporary Prevention of Comorbidities and Mortality

#### 2.3.1. Management of NAFLD and NASH

NAFLD, which may also be referred to as metabolic-associated fatty liver disease (MAFLD), is characterized by evidence of hepatic steatosis in the presence of at least one of the following: overweight/obesity, T2D, or evidence of metabolic dysregulation.^142,143^ Patients with NAFLD (or MAFLD) should optimally be identified before their condition progresses to NASH.^144,145^ NAFLD affects >70% of patients with T2D, who are also more susceptible to severe disease, including NASH.^146^

Screening for liver disease should be conducted annually among patients with obesity, dyslipidemia, polycystic ovary syndrome (PCOS), and/or T2D.^146,147^ Measurement of alanine transaminase (ALT) and aspartate transaminase (AST) is recommended, with the caveat that these tests lack sensitivity in detecting early fatty liver disease, which may be present even if results are normal, especially in individuals with insulin resistance.^148,149^ Standard ultrasonography can determine hepatic fat content but is not a good means of determining the presence or degree of hepatic fibrosis. The fibrosis 4 calculation (FIB-4) is easily calculated (based on platelets, ALT, AST, and age) and useful in estimating the risk of hepatic fibrosis that may be associated with NAFLD.^150^


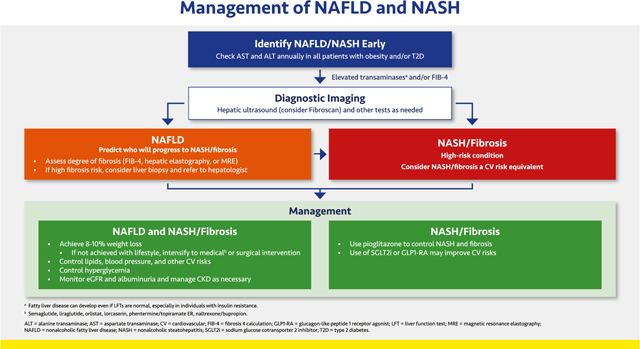


An abnormal ALT, AST, or FIB-4 may be followed up with ultrasound or MRI to assess hepatic fat content, and either Fibroscan or magnetic resonance elastography (MRE) used to determine the degree of hepatic fibrosis. Liver biopsy is the gold standard means of determining presence of NASH and is also useful for identifying other diseases that may cause or contribute to liver damage. The clinician should also consider and evaluate for other (or additional) potential etiologies of hepatic disease, including infectious hepatitis, hemochromatosis, and drug-related hepatotoxicity. Patients with or at high risk for fibrosis should be referred to a hepatologist.^146,147,150^

Management of patients with NAFLD who do not have NASH or significant fibrosis involves primarily lifestyle modification with management of other cardiovascular and renal risks as appropriate. Patients with overweight or obesity should strive for weight loss of at least 8% to 10%; if not achieved with lifestyle, medical or surgical weight loss interventions should be considered. Follow up (at least annually) should include regular reassessment for progression to more severe liver disease.

Care of patients with NASH or hepatic fibrosis requires a coordinated, multipronged approach that includes the lifestyle and risk factor recommendations for NAFLD as well as pioglitazone (regardless of presence of T2D) to address active steatohepatitis and reduce risk of progressive fibrosis.^146,151^ Mitigation of cardiovascular risks as discussed above is vital, including the use of statins and other agents to meet lipid and BP goals and smoking cessation. These patients may also benefit from SGLT2 inhibitor and/or a GLP1-RA.


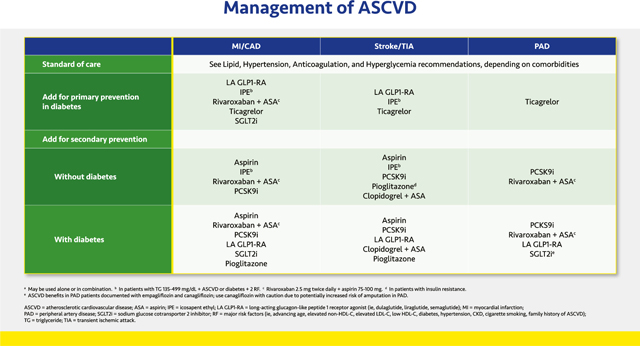


#### 2.3.2. Management of ASCVD

Management of ASCVD, including primary and secondary prevention of MI and CAD, stroke or TIA, and PAD, begins with controlling lipids, hypertension, and hyperglycemia as well as implementing anticoagulation therapy as appropriate for the individual patient. Once these conditions are controlled using standard therapies, additional risk reductions may be achieved with the other options shown in the slide.

For patients with T2D, primary prevention of MI and CAD with a GLP1-RA was demonstrated with dulaglutide, which reduced the relative risk of MACE by 12% after >5 years of treatment.^113^ Three GLP1-RAs—dulaglutide, liraglutide, and semaglutide—have demonstrated secondary prevention of MACE, with specific reductions in strokes. These agents may help prevent strokes in patients with T2D and established ASCVD.^111–113^ The SGLT2 inhibitors empagliflozin and canagliflozin have less robust data for MACE prevention but may still be considered to reduce risk of MI and CAD.^116–120^ Both GLP1-RAs and SGLT2 inhibitors may also be considered in patients with T2D and PAD to reduce the risks of cardiovascular events.

In patients with elevated triglycerides and established ASCVD or diabetes plus one other cardiovascular risk factor, IPE reduced the relative risk of a composite of cardiovascular death, nonfatal MI, nonfatal stroke, coronary revascularization, or unstable angina by 25%.^87^ In the diabetes subgroup, the relative risk reduction (RRR) was 23%.^87,152^ Reductions in each component of the composite endpoint were statistically significant in the overall population and the subgroup with diabetes.^87,152^ Based on these findings, IPE is recommended for primary prevention of MI, CAD, or stroke in patients with diabetes and for secondary prevention of these events in those with and without diabetes.

Treatment with rivaroxaban plus low-dose aspirin has been studied in patients with CAD or PAD with and without T2D. Overall, the combination reduced the risk of cardiovascular death, MI, or stroke by 24% vs aspirin alone, driven by a 22% decrease in cardiovascular death and a 42% reduction in stroke. There was also a 39% reduction in venous thromboembolism.^102^ Based on these findings, rivaroxaban plus low-dose aspirin is recommended for prevention of MI, stroke, cardiovascular death, and PAD events in those with and without diabetes who have CAD or PAD.

Aspirin alone is recommended for secondary prevention of ASCVD events in patients with and without diabetes, but because the risk of bleeding exceeds the benefits of aspirin therapy for patients with diabetes without a prior cardiovascular event, aspirin is generally not recommended for primary prevention in those with diabetes. However, aspirin should be considered in those with high cardiovascular risk.^31,153^

With ticagrelor, the RRR of MI, stroke, or cardiovascular death was 10% in patients with diabetes who had not had a prior cardiovascular event; individually, MI was reduced by 16% and stroke by 20%. In addition, major adverse limb events were reduced by 55%.^104^ Hence, ticagrelor is recommended for primary prevention of these events in patients with diabetes. The bleeding risks associated with antiplatelet and/or anticoagulant therapies should be considered before initiating these treatments.

The PCSK9 inhibitors are recommended for secondary prevention of MACE in patients with and without diabetes based on 15% RRR for cardiovascular death, MI, or stroke, as well as significant RRR in each component of the MACE composite, in separate trials.^67,68^

Pioglitazone reduced the relative risk of a composite of stroke or MI by 24% in patients with insulin resistance and a history of stroke or TIA (but not diabetes) and is therefore recommended for secondary prevention of stroke in this setting unless there are prevailing contraindications, such as HF.^128^ Indeed, this trial is exemplary in highlighting a precision medicine approach—i.e., using a drug in a population most likely to benefit. In patients with T2D, pioglitazone reduced the relative risk of the composite of all-cause mortality, MI, and stroke by 16%, although the primary endpoint of the trial (which included peripheral vascular disease outcomes) was not met.^127^

#### 2.3.3. Prevention and Management of Heart Failure

Prevention of HF begins with the same lifestyle interventions and risk factor control measures used for other conditions described in this guidance (see Section 2.1.1. Lifestyle Therapy; Section 2.2.2. Hypertension; Section 2.2.4. Antihyperglycemic Therapy; Section 2.3.2. ASCVD; Section 2.3.4. CKD). Patients with T2D with cardiovascular risk factors may be at risk of HF and should receive an angiotensin receptor blocker (ARB) or angiotensin-converting enzyme (ACE) inhibitor. An SGLT2 inhibitor and the addition of finerenone should be considered if the patient also has CKD.


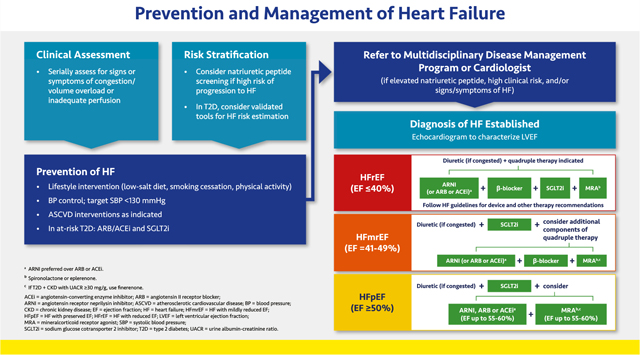


The clinical assessment of HF should begin with evaluation for signs or symptoms of congestion or inadequate perfusion, including dyspnea on exertion and decreased exercise tolerance. The American College of Cardiology Foundation (ACCF)/AHA define HF stages as extending from A (high risk for HF) to D (refractory HF). These definitions complement the traditional New York Health Association (NYHA) functional classifications, which are related to severity of symptoms and physical limitations in those with manifest HF.^154^ Natriuretic peptides (NT-proBNP and BNP) may be used to identify and stratify patients at risk for HF as well as to determine prognosis in those with manifest HF. BNP may increase in those taking an angiotensin receptor neprilysin inhibitor (ARNI; i.e., sacubitril/valsartan) because sacubitril inhibits enzymatic breakdown of BNP.^155^ Therefore, NT-proBNP is often preferred to monitor natriuretic peptides in ARNI-treated patients. For patients with T2D, validated tools (Machine Learning to Predict the Risk of Incident Heart Failure Hospitalization Among Patients With Diabetes [WATCH-DM] or Thrombolysis in Myocardial Infarction [TIMI] Risk Score for Heart Failure in Diabetes [TRS-HFDM]) are available for HF risk estimation.^156,157^ Patients with elevated natriuretic peptides, with high clinical risk, and/or those with signs or symptoms of HF should be referred to a cardiologist and/or multidisciplinary disease management program for prevention of HF or its progression.

HF is a clinical diagnosis based on signs and symptoms of congestion or inadequate perfusion. If HF is suspected, a two-dimensional echocardiogram coupled with Doppler flow studies is warranted to identify abnormalities of the myocardium, heart valves, and pericardium and evaluate left ventricular ejection fraction (LVEF).^158^ Approximately 31% of HF patients meet criteria for HF with reduced ejection fraction (HFrEF; EF ≤40%); 13% have mildly reduced ejection fraction (HFmrEF; EF 41% to 49%); and 56% have preserved ejection fraction (HFpEF; EF ≥50%).^159^ Among those hospitalized for HF, mortality risk is comparable with HFrEF, HFpEF, and HFmrEF, but the latter two are often underdiagnosed because clinicians may ascribe symptoms to obesity, aging, or other causes.^158,160–163^ Because HF is a progressive condition, it is important to identify and treat all conditions appropriately, including ongoing, intensive treatment of hypertension, obesity, and atrial fibrillation (if present).

Therapy for HFrEF should include ARNI, a beta blocker, a MRA (finerenone if the patient has T2D and CKD with albuminuria; otherwise spironolactone or eplerenone), and a diuretic (if congestion is present).^154,164^ An SGLT2 inhibitor should also be included regardless of the presence of T2D, as these agents have shown benefit in patients with and without diabetes.^40,119,120,124^ HF clinical practice guidelines for device-based recommendations should be followed for patients with HFrEF. Patients with HFmrEF should receive a diuretic (if congested) and a SGLT2 inhibitor and may also need the other components of quadruple therapy.

Patients with HFpEF should receive a SGLT2 inhibitor, based on the demonstrated benefits of empagliflozin and sotagliflozin in HFpEF.^123^ ARNI and RAS inhibitors may be appropriate for select patients with less than normal ejection fraction.^158,165^ Likewise, the nonsteroidal MRA finerenone may be considered for patients with HFpEF, T2D, and CKD (see Section 2.3.5. Comorbid Heart Failure and CKD). Diuretics may be considered for congestion.

#### 2.3.4. CKD Diagnosis and Treatment

CKD is defined as persistent eGFR <60 mL/min/1.73 m^2^ or UACR ≥30 mg/g.^166,167^ Diabetes and hypertension increase the risk of CKD, whereas CKD itself markedly increases risks of ASCVD, HF, arrhythmia, hypoglycemia, and premature mortality, and CKD also exacerbates comorbidities such as hypertension.^166,168^ When eGFR is reduced, CKD alters the menu of available drugs.^169^ Therefore, CKD alters the benefit-risk profiles of many important interventions.

Screening and diagnosis to define CKD is critical; eGFR *and* albuminuria should be measured at least annually.^4,7,30^ The new equation estimating GFR from serum creatinine does not include race, and an additional equation that adds serum cystatin C, are more precise than older methods.^170,171^ A single-voided (“spot”) urine measures albuminuria as ACR.

Lifestyle and goal-directed therapies form the critical foundation to reduce cardiovascular and CKD risk.^4,7^ Sodium restriction is mandatory because CKD impairs sodium excretion, exacerbating hypertension and HF. The maximum-tolerated dose of the RAS inhibitor should be used, consistent with clinical trials.^172^ A decrease in eGFR is expected with RAS inhibitor initiation, and a decrease as large as 30% is consistent with beneficial outcomes.


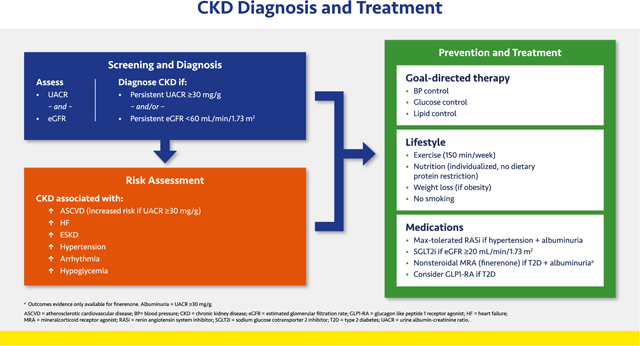


SGLT2 inhibitors reduce CKD progression and HF (30% to 40% RRR) as well as cardiovascular death.^173,174^ SGLT2 inhibitor trials demonstrate improved kidney outcomes (including reduced incidence of end-stage kidney disease [ESKD] among participants with T2D and CKD).^121,126^ The cardiac and renal benefits of SGLT2 inhibitors are independent of their glycemic effects and dose, and the class is standard of care for patients with T2D and CKD.^7,30,174^ Agents with proven benefits, such as canagliflozin (only in T2D) and dapagliflozin are indicated for patients with T2D with or without albuminuria or nondiabetic CKD with albuminuria (UACR ≥200 mg/g). They can be initiated at a eGFR as low as 20 mL/min/1.73 m^2^ and may be continued until dialysis (they are contraindicated in dialysis).^173,174^ An initial decrease in eGFR is expected with an SGLT2 inhibitor and per guidelines the agent should not be discontinued unless serious acute kidney injury is suspected.^175^ SGLT2 inhibitors are not approved for T1D. ^123,126,176^

GLP1-RAs reduce ASCVD events, for which patients with CKD are at high risk, but these agents have not been approved to slow CKD progression.^177^

The non-steroidal MRA finerenone reduced CKD progression and cardiovascular events (predominantly HF) in people with T2D and UACR ≥30 mg/g when added to standard of care, including a RAS inhibitor. Hyperkalemia occurred at higher rates with finerenone than placebo, but 99% of patients completed the trials.^178–180^ Finerenone can be added to standard of care treatment (including a RAS inhibitor and SGLT2 inhibitor) for people with T2D and CKD.

#### 2.3.5. Management of Comorbid Heart Failure and CKD

HF and CKD frequently intersect in clinical practice and have unique diagnostic, management, and monitoring considerations. Patients with comorbid HF and CKD face markedly elevated risks of clinical progression and mortality yet are often inadequately treated with disease-modifying therapies targeting each condition (“a risk-treatment paradox”).

Guideline-recommended HF therapies have been studied across a broad range of patients with comorbid CKD. The SGLT2 inhibitors have been studied and demonstrated to be safe and well-tolerated in patients with HF at eGFRs as low as 20 mL/min/1.73 m^2^.^173,174^ Other therapeutic classes, including ACE inhibitors, ARBs, ARNI, and steroidal MRAs (spironolactone and eplerenone) have been mostly studied at eGFR as low as 30 mL/min/1.73 m^2^. Although limited evidence exists for use of beta-blockers among those who require kidney-replacement therapy, no overt safety risks have been identified, and their use in those with HF may be considered.

From the novel class of nonsteroidal MRAs, finerenone has been shown to reduce cardiovascular and kidney disease events in patients with T2D and CKD with an eGFR as low as 25 mL/min/1.73 m^2^.^178,179^ In patients with comorbid HFmrEF or HFpEF, T2D, and CKD with albuminuria, the use of finerenone as the nonsteroidal MRA of choice appears reasonable. In other individuals with HFrEF, steroidal MRAs (spironolactone or eplerenone) are preferred if tolerated by the patient.

Hyperkalemia is frequently observed with ACE inhibitors, ARBs, and MRAs, especially if eGFR is ≤45 mL/min/1.73 m^2^. This condition often limits up-titration or use of evidence-based doses of these therapies in HF and CKD.^181^ The concomitant use of an ARNI or SGLT2 inhibitor has been shown to lower risks of hyperkalemia related to both classes of MRA, and combination use may promote treatment persistence in practice.^182^ The use of potassium binders such as patiromer and sodium zirconium cyclosilicate may be considered to facilitate use of these therapies among patients who experience therapy-related hyperkalemia.

In HF, many vasoconstrictor peptides that maintain GFR are elevated. Many therapies used in both HF and CKD lower intra-glomerular pressures, and treatment initiation may result in acute eGFR decline, especially if the patient has volume depletion. This eGFR decline is not associated with renal safety signals in clinical trials with or without HF.^183,184^ As such, this hemodynamic effect should not prompt treatment discontinuation or de-escalation in most cases. If eGFR declines by more than 30% within a week of treatment initiation, and volume depletion is excluded, alternative etiologies should be evaluated and concomitant diuretic adjustments may be considered.

Monitoring of UACR and natriuretic peptides may be considered to evaluate disease progression. Declines in these biomarkers with therapy have been associated with improved clinical outcomes.^179^ Specifically, a sustained reduction of ≥30% in albuminuria is considered a surrogate for good renal outcome.

#### 2.3.6 Summary of Medications for Diabetes, Lipid, and Kidney Disorders

Along with lifestyle recommendations, pharmacotherapy is usually necessary to address the multiple cardiorenal and metabolic defects of patients with and without T2D. The table provides a brief summary of the most common benefits, concerns, and contraindications for medication classes commonly used for patients with T2D, hyperlipidemia, HF, and/or CKD. Treatment decisions should be made based on good clinical judgement, individual patients’ needs and characteristics, product indications and restrictions, clinical practice guidelines, and other relevant factors.


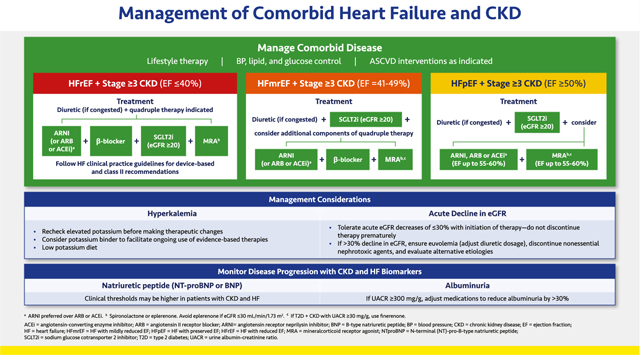



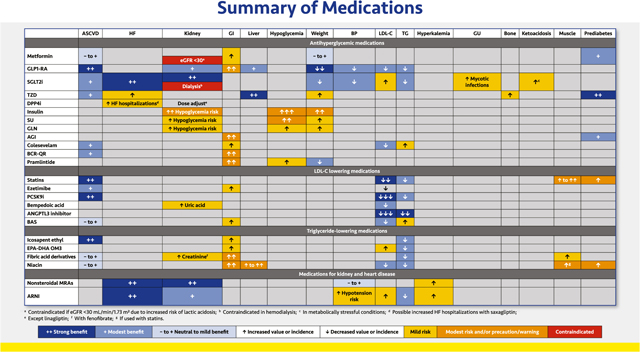


##### 2.3.6.1. Antihyperglycemic agents.

Thorough reviews of the attributes of antihyperglycemic classes can be found elsewhere.^5,129^ Compared with sulfonylureas, metformin is associated with increased cardiovascular safety and more durable antihyperglycemic effects. This agent does not promote hypoglycemia and may induce mild weight loss. It should not be initiated if eGFR is <45 mL/min/1.73 m^2^, but established therapy may be continued with stable eGFR ≥30 mL/min/1.73 m^2^.^4,185^ Vitamin B12 deficiency can develop, and supplementation may be needed to address associated anemia and/or peripheral neuropathy.^186^ Among patients with prediabetes, metformin may delay progression to T2D.^187^

GLP1-RAs yield robust glycemic reductions as well as decreases in weight, BP, and lipids and carry a low risk of hypoglycemia. Most GLP1-RAs are given as injections (daily or weekly); currently one oral formulation is available. Dulaglutide, liraglutide, and injectable semaglutide have been shown to improve cardiovascular outcomes.^111–113^ Gastrointestinal side effects can be mitigated by careful, slow dose titration. GLP1-RAs are contraindicated in patients with a personal or family history of MCT or MEN type 2, and caution should be exercised in patients with a history of acute pancreatitis. Exenatide is contraindicated if eGFR is <30 mL/min/1.73 m^2^, and renal function should be monitored with all GLP1-RAs, especially in patients with nausea and possible dehydration.^188^

SGLT2 inhibitors reduce glycemia, weight, and BP. The class reduces HF hospitalizations and improves kidney function; some SGLT2 inhibitors have been shown to reduce the risk of other cardiovascular events.^115–118,121,122^ Dapagliflozin and empagliflozin have been shown to improve HF and/or CKD outcomes in patients without diabetes.^123,176^ The cardiorenal benefits of SGLT2 inhibitors are independent of glucose lowering, and the class may be used to an eGFR <20 mL/min/1.73 m^2^. However, glucose reductions diminish as eGFR declines, and these agents are contraindicated in dialysis patients.^188^ Adverse effects include increased risk of genital mycotic infections and LDL-C increases. Necrotizing fasciitis of the perineum is a rare complication. In patients with T1D and insulinopenic T2D, concomitant use of SGLT2 inhibitors and insulin may increase DKA risk.^189^

The TZD pioglitazone may improve cardiovascular outcomes and NAFLD/NASH.^127,128,146,151^ TZDs have robust A1C-lowering effects and carry a low risk of hypoglycemia but increase the risk of weight gain, edema, HF exacerbation, and osteoporotic fractures.^5,129,190^ Side effects can be mitigated by utilizing smaller doses (pioglitazone 15 or 30 mg/day). Concomitant use of SGLT2 inhibitors and/or diuretic therapy can mitigate fluid retention, whereas insulin may aggravate fluid retention.

DPP4 inhibitors prolong the half-life of endogenous incretin hormones but are less efficacious in A1C reduction than GLP-1 RAs. They also lack the weight loss and cardiovascular benefits of GLP1-RAs. A possible increase in HF hospitalizations with saxagliptin has not been shown with other DPP4 inhibitors. Dosage adjustments in CKD are required for all DPP4 inhibitors except linagliptin^.5,129^

Although insulin has the greatest glucose-lowering potential of all antihyperglycemic agents, in practice insulin is limited by the risk of hypoglycemia. Weight gain is also common, due to both the anabolic effects of the hormone and to increased caloric consumption in fear of (or as a treatment for) hypoglycemia. In T2D, insulin (usually as basal insulin) should be started when glucose cannot be controlled with other agents, and the insulin regimen should be intensified as the disease progresses (see detailed reviews of insulin therapy in T2D).^5,129^ CGM (or structured SMBG for patients without access to CGM) is essential for patients on insulin therapy to ensure optimal dosing and safety. Glucagon should be prescribed for all patients on insulin.

Sulfonylureas elicit relatively potent glycemic reductions, but side effects may include weight gain and hypoglycemia. Glinides are short-acting insulin secretagogues that may not be as efficacious as sulfonylureas, but their shorter half-life and meal-time usage is associated with a lower risk of hypoglycemia.^5,129^

AGIs are used relatively infrequently in the US. Taken three times daily, they modestly reduce A1C levels but are associated with various gastrointestinal adverse effects. In prediabetes, these agents delayed progression to T2D.^38^ Liver disease in patients with CKD treated with AGIs has been reported.^5,129^

The bile acid sequestrant colesevelam has a modest glucose-lowering effect in addition to lowering LDL-C, but its use may be limited by gastrointestinal symptoms and triglyceride elevations in patients with pre-existing hypertriglyceridemia.^5,129,191^

Bromocriptine-quick release (BCR-QR) reduces A1C without hypoglycemia or weight gain and may improve cardiovascular outcomes.^192^ The major adverse effects include nausea and orthostatic hypotension, which can be mitigated by careful dose titration.^5,129^

Pramlintide is an injectable amylin analog agent administered with insulin prior to meals to slow gastric emptying. It may contribute to hypoglycemia due to its co-administration with insulin. Insulin dosages need to be reduced when pramlintide is initiated and titrated.^5,129^

##### 2.3.6.2. LDL-C lowering drugs.

Comprehensive reviews of LDL-C and triglyceride-lowering agents are available elsewhere.^6,59^ Statins, the mainstay of lipid-lowering therapy, reduce both LDL-C and triglycerides and have demonstrated consistent reductions in ASCVD in numerous CVOTs.^6,59^ Myopathy and in rare cases rhabdomyolysis are the primary adverse effects of concern. Worsening glucose tolerance and hastened development of T2D may also occur, but these effects are outweighed by the ASCVD benefits.^193,194^

Ezetimibe, a cholesterol absorption inhibitor, is often used adjunctively with statins or other non-statin agents to further lower LDL-C.^6,59^ In Improved Reduction of Outcomes: Vytorin Efficacy International Trial (IMPROVE-IT), the cardiovascular benefit was additive to baseline statin therapy, especially among patients with T2D. ^69^ Ezetimibe is generally well tolerated but can cause some GI discomfort.

PCSK9 inhibitors have been studied mainly in combination with statins and yield robust LDL-C reductions with substantial reductions in ASCVD risk.^6,59^ These agents are injected bimonthly or monthly.

Bempedoic acid is an oral agent that lowers LDL-C by inhibiting ATP citrate lyase, a precursor of cholesterol synthesis that is available alone and in a single-pill combination with ezetimibe. It is associated with increases in uric acid and gout, and tendon rupture is a rare complication.^6^

An ANGPTL3 inhibitor, evinacumab is administered by once monthly infusion to treat homozygous familial hypercholesterolemia. At this time, this therapy is generally given only by lipid specialists.

Bile acid sequestrants were used more frequently before statins became available. These agents may cause significant gastrointestinal distress and can interfere with the absorption of other medications. In addition, they may modestly increase triglyceride levels. CVOTs involving small numbers of patients have shown a neutral to mild benefit.^6^

##### 2.3.6.3. Triglyceride-lowering drugs.

IPE is a purified formulation of EPA that reduces triglycerides and also confers cardiovascular benefits that may be mediated by anti-inflammatory, antiplatelet, antioxidant, and possibly other mechanisms beyond triglyceride reductions. IPE is associated with gastrointestinal adverse effects, increased bleeding, and atrial fibrillation.^6^

Combination EPA/DHA formulations reduce triglycerides levels but do not appear to reduce cardiovascular risk. Adverse effects include gastrointestinal intolerance. Prescription strength formulations of EPA/DHA are preferred because over-the-counter formulations may have impurities; may be contaminated with saturated, polyunsaturated, and trans fats; or may not contain consistent quantities of EPA/DHA.^6^

Fibrates may be the most potent triglyceride-lowering class, but these agents are associated with LDL-C increases, and fenofibrate may also increase creatinine. The risk of myopathy is increased when some fibrates are combined with certain statins; gemfibrozil is contraindicated with simvastatin.^6^

Niacin reduces triglycerides and may also modestly reduce LDL-C. Adverse effects include flushing, pruritis, nausea, and glucose increases, as well as possibly increased myopathy when combined with statins. Hepatotoxicity may occur, especially in patients taking over-the-counter niacin supplements.^6^

##### 2.3.6.4. Nonsteroidal MRA.

Finerenone is currently the only nonsteroidal MRA available. This agent blocks sodium reabsorption through the mineralocorticoid receptor and also reduces overactivation of this receptor in the kidney, heart, and blood vessels.^195,196^ In clinical trials, it reduced CKD progression, ESKD, HF hospitalization, and other cardiovascular outcomes in patients with CKD and T2D.^178^ It is associated with an increased risk of hyperkalemia. It is not recommended if eGFR is <25 mL/min/1.73 m^2^, and it is contraindicated in patients with adrenal insufficiency.

##### 2.3.6.5. ARNI.

The ARNI sacubitril/valsartan is a single-pill combination of a neprilysin inhibitor (sacubitril) and an ARB (valsartan). In patients with HFrEF, sacubitril/valsartan reduced BP and the risk of death and HF hospitalizations; it may also help preserve kidney function.^197–199^ Modest decreases in triglycerides and increases in HDL-C and LDL-C have been reported.^200^ Sacubitril/valsartan may increase the risk of hypotension, hyperkalemia, and acute renal failure, and it should not be used with other RAS inhibitors, including ARBs, ACE inhibitors, or aliskiren.

### 2.4. Future outlook and conclusions

Our goal has been to bridge the gap between separate, individual specialties and make integrated recommendations that can be directly applied to complex individual patients within primary care or specialty practices. We recognize the unique roles and overwhelming amount of data primary care physicians and specialists must incorporate into their clinical practice. We can expect that future studies will continue to add further tools and expand our understanding of the intersection between the diagnosis, pathophysiology, and treatment of patients with underlying diabetes, cardiorenal, and/or metabolic (DCRM) disorders. Therefore, we hope these practice recommendations designed for the nonexpert will be an initial framework on which to build comprehensive treatment plans that will help clinicians provide optimal care, leading to improved outcomes for our patients. As more medications and CVOTs in the fields of diabetes, NAFLD, NASH, and obesity become available, we anticipate updating and extending the DCRM. We also look forward to the international community joining us and helping make these recommendations even more universal.

## Supplementary Material

Supplementary Material

Supplementary Tables 1 and 2

## Figures and Tables

**Table 1:** “Know your numbers” suggested plain-language communication points to patients.

Parameter	What it tells us	What’s normal	Whaťs risky	The direction we want it to go^a^
General health (all patients)				
BMI	Whether your weight puts you at risk for other diseases. BMI is your weight (in kilograms) divided by your height (in meters)	18 to 25	30 or more	Lower
Waist circumference	A way of measuring how much fat you have around your stomach area; too much puts you at risk for other diseases	Women ≤88 cm (35 in); men ≤102 cm (40 in)	More than these	Lower
BP	The amount of pressure your blood puts against the walls of your blood vessels (like the water in a hose)	Less than 120 over 80	More than 140 over 90	Lower
HDL-C	How much “good” cholesterol you have, which helps keep the blood flowing in your body	More than 50	Less than 40	Higher
Triglycerides	How much fat is in your blood	Less than 100	More than 135	Lower
LDL-C	How much “bad” cholesterol you have; too much can clog up your blood vessels	Less than 100	More than 55, 70, or 100^b^	Lower
Non-HDL-C	Total cholesterol minus HDL-C (“good” cholesterol)	Less than 130	More than 85, 100, or 130^b^	Lower
Diabetes				
A1C	How well your diabetes is controlled overall	Less than 5.7	More than 6.5 or 7 or 7.5^c^	Lower
FPG	How much sugar is in your blood when you haven’t eaten for 8 h, such as in the morning before breakfast	More than 70 and less than 100	Less than 70 and more than 140	Stay between 70 and 140
TIR	The percentage of time each day your blood sugar is well controlled	100%	<70%	Longer (more time)
Diabetes and CKD				
eGFR	How well your kidneys are working	More than 90	Less than 60	Higher (or at least stay the same)
UACR	How much protein is in your urine, which tells us if your kidneys are damaged	Less than 30	More than 300	Lower (or at least stay the same)

Abbreviations: A1C, hemoglobin A1C; BMI, body mass index; BP, blood pressure; eGFR, estimated glomerular filtration rate; HDL-C, high density lipoprotein cholesterol; LDL-C, low density lipoprotein cholesterol; TIR, time in range; UACR, urine albumin-creatinine ratio.

a Assumes patient’s levels are abnormal.

b Depends on the patient’s individual comorbidities; see Section 2.2.1. Lipids.

c Depends on patient’s individual characteristics; see Section 2.2.4. Antihyperglycemic Therapy.
