# Supplementary material for: DCRM Multispecialty Practice Recommendations for the management of diabetes, cardiorenal, and metabolic diseases
Source: J Diabetes Complications. Author manuscript; Available in PMC 2022 Dec 30. (PMC9803322; doi:10.1016/j.jdiacomp.2021.108101)
Supplement: Supplementary Material [file NIHMS1853694-supplement-Supplementary_Material.pdf]

Supplementary Appendix for

DCRM Multispecialty Practice Recommendations for the Management of Diabetes, Cardiorenal, and Metabolic Diseases

Yehuda Handelsman (Chair), John E. Anderson, George L. Bakris, Christie M. Ballantyne, Joshua A. Beckman, Deepak L. Bhatt, Zachary T. Bloomgarden, Biykem Bozkurt, Matthew J. Budoff, Javed Butler, Samuel Dagogo-Jack, Ian H. de Boer, Ralph A. DeFronzo, Robert H. Eckel, Daniel Einhorn, Vivian A. Fonseca, Jennifer B. Green, George Grunberger, Chris Guerin, Silvio E. Inzucchi, Paul S. Jellinger, Mikhail N. Kosiborod, Pamela Kushner, Norman Lepor, Christian W. Mende, Erin D. Michos, Jorge Plutzky, Pam R. Taub, Guillermo E. Umpierrez, Muthiah Vaduganathan, Matthew R. Weir

Table S1. Risk-based LDL-C goals from the ACC/AHA, AACE, and ESC/EAS

LDL-C target	Society	Society risk category
Consider <40 mg/dL (<1.0 mmol/L)	ESC/EAS[1]	Very high risk-plus
<55 mg/dL (<1.4 mmol/L)	AACE[2]	Extreme risk
	ESC/EAS[1]	Very high risk
<70 mg/dL (<1.8 mmol/L)	AACE[2]	Very high risk
	ESC/EAS[1]	High risk
	ACC/AHA[3]	Very high risk

AACE, American Association of Clinical Endocrinologists; ACC, American College of Cardiology; AHA, American Heart Association; EAS, European Atherosclerotic Society; ESC, European Society of Cardiology; LDL-C, low-density lipoprotein cholesterol.

Table S2. ASCVD risk calculators

Name	Web address
Multiethnic Study of Atherosclerosis (MESA) CHD risk estimator[4]	https://www.mesa-nhlbi.org/CAC-Tools.aspx
Framingham Risk Equation[5]	https://framinghamheartstudy.org/fhs-risk-functions/cardiovascular-disease-10-year-risk/
Reynold's Risk Score[6]	http://www.reynoldsriskscore.org
United Kingdom Prospective Diabetes Study (UKPDS) Risk Engine (for patients with diabetes)[7]	https://www.dtu.ox.ac.uk/riskengine/
ACC/AHA pooled-cohort ASCVD Risk Estimator[8]	www.cvriskcalculator.com
Building, Relating, Assessing, and Validating Outcomes (BRAVO) risk engine[9]	http://www.bravo4health.com
Systematic Coronary Risk Evaluation 2 (SCORE2)[10]	https://www.escardio.org/Education/Practice-Tools/CVD-prevention-toolbox/SCORE-Risk-Charts

References

1. Mach F, Baigent C, Catapano AL, et al. 2019 ESC/EAS Guidelines for the management of dyslipidaemias: lipid modification to reduce cardiovascular risk. *Eur Heart J.* 2020;41(1): 111-188.
2. Jellinger PS, Handelsman Y, Rosenblit PD, et al. American Association of Clinical Endocrinologists and American College of Endocrinology guidelines for management of dyslipidemia and prevention of cardiovascular disease. *Endocr Pract*. 2017;23(Suppl 2): 1-87.
3. Grundy SM, Stone NJ, Bailey AL, et al. 2018 AHA/ACC/AACVPR/AAPA/ABC/ ACPM/ADA/AGS/APhA/ASPC/NLA/PCNA guideline on the management of blood cholesterol: a report of the American College of Cardiology/American Heart Association Task Force on Clinical Practice Guidelines. *J Am Coll Cardiol*. 2019;73(24): e285-e350.
4. McClelland RL, Jorgensen NW, Budoff M, et al. 10-Year coronary heart disease risk prediction using coronary artery calcium and traditional risk factors: derivation in the MESA (Multi-Ethnic Study of Atherosclerosis) with validation in the HNR (Heinz Nixdorf Recall) study and the DHS (Dallas Heart Study). *J Am Coll Cardiol.* 2015;66(15): 1643-1653.
5. Lloyd-Jones DM, Wilson PW, Larson MG, et al. Framingham risk score and prediction of lifetime risk for coronary heart disease. *Am J Cardiol.* 2004;94(1): 20-24.
6. Ridker PM, Buring JE, Rifai N, Cook NR. Development and validation of improved algorithms for the assessment of global cardiovascular risk in women: the Reynolds Risk Score. *JAMA.* 2007;297(6): 611-619.
7. Stevens RJ, Kothari V, Adler AI, Stratton IM. The UKPDS risk engine: a model for the risk of coronary heart disease in type II diabetes (UKPDS 56). *Clin Sci (Lond).* 2001;101(6): 671-679.
8. Goff DC, Jr., Lloyd-Jones DM, Bennett G, et al. 2013 ACC/AHA guideline on the assessment of cardiovascular risk: a report of the American College of Cardiology/American Heart Association Task Force on Practice Guidelines. *Circulation.* 2013;129(25 suppl 2): S49-73.
9. Shao H, Fonseca V, Stoecker C, Liu S, Shi L. Novel Risk Engine for Diabetes Progression and Mortality in USA: Building, Relating, Assessing, and Validating Outcomes (BRAVO). *PharmacoEconomics.* 2018;36(9): 1125-1134.
10. SCORE2 working group, ESC Cardiovascular risk collaboration. SCORE2 risk prediction algorithms: new models to estimate 10-year risk of cardiovascular disease in Europe. *Eur Heart J.* 2021;42(25): 2439-2454.

Declaration of Competing Interests

Y. Handelsman reports research grants and consultant and speaker honoraria from Amarin, Amgen, Applied Therapeutic, Astra Zeneca, Bayer, Boehringer Ingelheim, Corcept, Esperion, Ionis, Merck, Merck-Pfizer, Novartis, Novo Nordisk, Sanofi, and Vertis.

J.E. Anderson reports consulting fees or speaker honoraria from Eli Lilly, Novo Nordisk, Abbott Diabetes, Sanofi, Boehringer Ingelheim, Bayer, Gelesis, Astra Zeneca, and Alfa Sigma.

G.L. Bakris reports consulting fees from Merck, Bayer, KBP Biosciences, Ionis, Alnylam, Astra Zeneca, Quantum Genomics, Horizon, Novo Nordisk, and DiaMedica Therapeutics, and his institution has received research grants from Bayer, Vascular Dynamics, Novo Nordisk, and Janssen.

C.M. Ballantyne reports consulting fees from Abbott Diagnostics, Althera, Amarin, Amgen, Arrowhead, Astra Zeneca, Denka Seiken, Esperion, Genentech, Gilead, Illumina, Matinas BioPharma Inc, Merck, New Amsterdam, Novartis, Novo Nordisk, Pfizer, Regeneron, Roche Diagnostic, and Sanofi-Synthelabo; his institution received research grants or support from Abbott Diagnostic, Akcea, Amgen, Arrowhead, Esperion, Ionis, Novartis, Regeneron, Roche Diagnostic, the National Institutes of Health, the American Heart Association, and the American Diabetes Association; and he has served on the boards of the National Lipid Association and the American Society for Clinical Pathology.

J.A. Beckman reports consulting fees from Amgen and JanOne; service on a data safety monitoring or advisory board for Novartis, Janssen, and Kowa; and service as board president for VIVA Physicians.

D.L. Bhatt discloses the following relationships: Advisory Board: Boehringer Ingelheim, Cardax, CellProthera, Cereno Scientific, Elsevier Practice Update Cardiology, Janssen, Level Ex, Medscape Cardiology, MyoKardia, NirvaMed, Novo Nordisk, PhaseBio, PLx Pharma, Regado Biosciences, Stasys; Board of Directors: Boston VA Research Institute, Society of Cardiovascular Patient Care, TobeSoft; Chair: Inaugural Chair, American Heart Association Quality Oversight Committee; Data Monitoring Committees: Baim Institute for Clinical Research (formerly Harvard Clinical Research Institute, for the PORTICO trial, funded by St. Jude Medical, now Abbott), Boston Scientific (Chair, PEITHO trial), Cleveland Clinic (including for the ExCEED trial, funded by Edwards), Contego Medical (Chair, PERFORMANCE 2), Duke Clinical Research Institute, Mayo Clinic, Mount Sinai School of Medicine (for the ENVISAGE trial, funded by Daiichi Sankyo), Novartis, Population Health Research Institute; Honoraria: American College of Cardiology (Senior Associate Editor, Clinical Trials and News, ACC.org; Chair, ACC Accreditation Oversight Committee), Arnold and Porter law firm (work related to Sanofi/Bristol-Myers Squibb clopidogrel litigation), Baim Institute for Clinical Research (formerly Harvard Clinical Research Institute; RE-DUAL PCI clinical trial steering committee funded by Boehringer Ingelheim; AEGIS-II executive committee funded by CSL Behring), Belvoir Publications (Editor in Chief, Harvard Heart Letter), Canadian Medical and Surgical Knowledge Translation Research Group (clinical trial

steering committees), Cowen and Company, Duke Clinical Research Institute (clinical trial steering committees, including for the PRONOUNCE trial, funded by Ferring Pharmaceuticals), HMP Global (Editor in Chief, Journal of Invasive Cardiology), Journal of the American College of Cardiology (Guest Editor; Associate Editor), K2P (Co-Chair, interdisciplinary curriculum), Level Ex, Medtelligence/ReachMD (CME steering committees), MJH Life Sciences, Piper Sandler, Population Health Research Institute (for the COMPASS operations committee, publications committee, steering committee, and USA national co-leader, funded by Bayer), Slack Publications (Chief Medical Editor, Cardiology Today's Intervention), Society of Cardiovascular Patient Care (Secretary/Treasurer), WebMD (CME steering committees); Other: Clinical Cardiology (Deputy Editor), NCDR-ACTION Registry Steering Committee (Chair), VA CART Research and Publications Committee (Chair); Research Funding: Abbott, Afimmune, Amarin, Amgen, AstraZeneca, Bayer, Boehringer Ingelheim, Bristol-Myers Squibb, Cardax, CellProthera, Cereno Scientific, Chiesi, CSL Behring, Eisai, Ethicon, Faraday Pharmaceuticals, Ferring Pharmaceuticals, Forest Laboratories, Fractyl, Garmin, HLS Therapeutics, Idorsia, Ironwood, Ischemix, Janssen, Javelin, Lexicon, Lilly, Medtronic, MyoKardia, NirvaMed, Novartis, Novo Nordisk, Owkin, Pfizer, PhaseBio, PLx Pharma, Regeneron, Roche, Sanofi, Stasys, Synaptic, The Medicines Company, 89Bio; Royalties: Elsevier (Editor, Cardiovascular Intervention: A Companion to Braunwald's Heart Disease); Site Co-Investigator: Abbott, Biotronik, Boston Scientific, CSI, St. Jude Medical (now Abbott), Philips, Svelte; Trustee: American College of Cardiology; Unfunded Research: FlowCo, Merck, Takeda.

Z.T. Bloomgarden reports stock in Johnson & Johnson and Novartis.

B. Bozkurt reports consulting fees from Amgen, Baxter Healthcare Corporation, Bristol Myers Squibb, Relypsa, sanofi-aventis, and scPharmaceuticals; service on an advisory or data safety monitoring board for Abbott Vascular and Liva Nova; and service as President of the Heart Failure Society of America.

M.J. Budoff reports honoraria from Astra Zeneca, Boehringer Ingelheim, and Novo Nordisk and a grant from Novo Nordisk.

J. Butler reports consulting fees from Abbott, Adrenomed, Amgen, Applied Therapeutics, Array, Astra Zeneca, Bayer, Boehringer Ingelheim, CVRx, G3 Pharma, Impulse Dynamics, Innolife, Janssen, LivaNova, Luitpold, Medtronic, Merck, Novartis, NovoNordisk, Relypsa, Sequana Medical, and Vifor and honoraria from Novartis, Boehringer Ingelheim/Lilly, Astra Zeneca, and Janssen.

S Dagogo-Jack reports consulting fees from Astra Zeneca, Bayer, Boehringer Ingelheim, Janssen, Medtronic, Merck, and Sanofi and that his institution has received research funding from AstraZeneca, Boehringer Ingelheim, and Novo Nordisk.

I. H. de Boer reports grants from the National Institute of Diabetes and Digestive and Kidney Diseases; National Heart, Lung, and Blood Institute; and the Juvenile Diabetes Research Foundation; consulting fees from Astra Zeneca, Bayer, Boehringer Ingelheim, Cyclerion Therapeutics, George Clinical, Gilead, Goldfinch Bio, Ironwood; honoraria from Medscape; and receipt of equipment, materials, drugs, medical writing, gifts, or other services from DexCom.

R.A. DeFronzo reports honoraria from Astra Zeneca; travel reimbursement from Astra Zeneca, Boehringer Ingelheim, Intarcia, and Novo Nordisk; and service on a data safety monitoring board or advisory board for Astra Zeneca, Boehringer Ingelheim, Intarcia, and Novo Nordisk.

R.H. Eckel reports consulting fees from Amarin, Up To Date, KOWA, Novo Nordisk, and WorldWide; honoraria from Tarsus, Inc., and the American College of Cardiology; travel reimbursement from the American Diabetes Association; and service on the board of the American Diabetes Association.

D. Einhorn reports honoraria from Novo Nordisk, Eli Lilly, Boehringer Ingelheim, Abbott, and Corcept Pharma; service on an advisory board for Intuity Medical; and stock or stock options in Corcept Pharma and Epitracker/Seraphina.

V.A. Fonseca reports consulting fees and honoraria from Takeda, Novo Nordisk, sanofi-aventis, Bayer Abbott, Astra Zeneca, Intarcia, and Asahi; payment for expert testimony from Boehringer Ingelheim; stock options or stock from Mellitus Health, BRAVO4Health, and Amgen; and his institution has received grants from Fractyl and Jaguar Gene Therapy.

J.B. Green reports grants from Boehringer Ingelheim/Lilly, Merck, Sanofi/Lexicon, Astra Zeneca, and Roche; consulting fees from Boehringer Ingelheim/Lilly, Sanofi/Lexicon, Pfizer, Bayer, Hawthorne Effect/Omada, and Astra Zeneca; and service on a data monitoring safety board for Novo Nordisk.

G. Grunberger reports honoraria from Eli Lilly, Novo Nordisk, and Abbott Diabetes; service on a data safety monitoring board for the National Institutes of Health and the board of directors for Children With Diabetes.

C. Guerin reports service on a board or committee for the Clinical Association of California Endocrinologists and the American Association of Clinical Endocrinologists.

S.E. Inzucchi reports consulting fees or other support from Astra Zeneca, Boehringer Ingelheim, Novo Nordisk, Lexicon, Merck, Pfizer, vTv Therapeutics, Esperion, and Abbott; he has given lectures sponsored by Astra Zeneca and Boehringer Ingelheim.

P.S. Jellinger reports honoraria from Esperion and Amgen.

M.N. Kosiborod reports grants from Astra Zeneca and Boehringer-Ingelheim and consulting fees from Amgen, Applied Therapeutics, Astra Zeneca, Bayer, Boehringer-Ingelheim, Eli Lilly, Esperion Therapeutics, Janssen, Merck (diabetes & cardiovascular), Novo Nordisk, Sanofi, and Vifor Pharma.

P. Kushner has no interests or relationships to report.

N. Lepor reports honoraria from Amgen, Boehringer Ingelheim, Esperion, Lilly, and Novartis and stock or stock options in Amarin.

C.W. Mende reports honoraria from Bayer, Boehringer Ingelheim, Lilly, Astra Zeneca, and Janssen; a patent for fatty kidney disease; and service on a data safety monitoring or advisory board for Boehringer Ingelheim and Astra Zeneca.

E.D. Michos reports consulting fees from Astra Zeneca, Esperion, Amarin, Bayer, Novartis, and Novo Nordisk.

J. Plutzky reports consulting fees from Alnylam, Amgen, Novo Nordisk, PharmApprove, and Vivus; service on the American Heart Association Diabetes Committee and the Sarnoff Scientific Committee; his institution has received grants from Boehringer Ingelheim and Novartis

P.R. Taub reports grants from the National Institutes of Health, the American Heart Association, and the Federal Emergency Management Agency; consulting fees from Amarin, Amgen, Bayer, Boehringer-Ingelheim, Novartis, Novo-Nordisk, Sanofi, Esperion Therapeutics, Medtronic; stock or stock options in Epirium Bio.

G.E. Umpierrez reports his institution has received grants from Novo Nordisk, Dexcom, Astra Zeneca, and Sanofi.

M. Vaduganathan reports consulting fees from American Regent, Amgen, AstraZeneca, Bayer AG, Baxter Healthcare, Boehringer Ingelheim, Cytokinetics, Lexicon Pharmaceuticals, Relypsa, and Roche Diagnostics; honoraria from Novartis and Roche Diagnostics; service on boards for Novartis and Galmed; and his institution has received grants from Amgen, AstraZeneca, Boehringer Ingelheim, and Roche Diagnostics.

M.R. Weir reports grants from the NIDDK and NHLBI and consulting fees from Astra Zeneca, Bayer, Vifor, Akebia, Novo Nordisk, Boehringer Ingelheim, Janssen, and Merck
