# Supplementary material for: DCRM Multispecialty Practice Recommendations for the management of diabetes, cardiorenal, and metabolic diseases
Source: J Diabetes Complications. Author manuscript; Available in PMC 2022 Dec 30. (PMC9803322; doi:10.1016/j.jdiacomp.2021.108101)
Supplement: Supplementary Tables 1 and 2 [file NIHMS1853694-supplement-Supplementary_Tables_1_and_2.pdf]

Tables S1 and S2

DCRM Multispecialty Practice Recommendations for the Management of Diabetes, Cardiorenal, and Metabolic Diseases

Yehuda Handelsman (Chair), John E. Anderson, George L. Bakris, Christie M. Ballantyne, Joshua A. Beckman, Deepak L. Bhatt, Zachary T. Bloomgarden, Biykem Bozkurt, Matthew J. Budoff, Javed Butler, Samuel Dagogo-Jack, Ian H. de Boer, Ralph A. DeFronzo, Robert H. Eckel, Daniel Einhorn, Vivian A. Fonseca, Jennifer B. Green, George Grunberger, Chris Guerin, Silvio E. Inzucchi, Paul S. Jellinger, Mikhail N. Kosiborod, Pamela Kushner, Norman Lepor, Christian W. Mende, Erin D. Michos, Jorge Plutzky, Pam R. Taub, Guillermo E. Umpierrez, Muthiah Vaduganathan, Matthew R. Weir

Table S1. Risk-based LDL-C goals from the ACC/AHA, AACE, and ESC/EAS

LDL-C target	Society	Society risk category
Consider <40 mg/dL (<1.0 mmol/L)	ESC/EAS[1]	Very high risk-plus
<55 mg/dL (<1.4 mmol/L)	AACE[2]	Extreme risk
	ESC/EAS[1]	Very high risk
<70 mg/dL (<1.8 mmol/L)	AACE[2]	Very high risk
	ESC/EAS[1]	High risk
	ACC/AHA[3]	Very high risk

AACE, American Association of Clinical Endocrinologists; ACC, American College of Cardiology; AHA, American Heart Association; EAS, European Atherosclerotic Society; ESC, European Society of Cardiology; LDL-C, low-density lipoprotein cholesterol.

Table S2. ASCVD risk calculators

**Name**	**Web address**
Multiethnic Study of Atherosclerosis (MESA) CHD risk estimator[4]	https://www.mesa-nhlbi.org/CAC-Tools.aspx
Framingham Risk Equation[5]	https://framinghamheartstudy.org/fhs-risk-functions/cardiovascular-disease-10-year-risk/
Reynold's Risk Score[6]	http://www.reynoldsriskscore.org
United Kingdom Prospective Diabetes Study (UKPDS) Risk Engine (for patients with diabetes)[7]	https://www.dtu.ox.ac.uk/riskengine/
ACC/AHA pooled-cohort ASCVD Risk Estimator[8]	www.cvriskcalculator.com
Building, Relating, Assessing, and Validating Outcomes (BRAVO) risk engine[9]	http://www.bravo4health.com
Systematic Coronary Risk Evaluation 2 (SCORE2)[10]	https://www.escardio.org/Education/Practice-Tools/CVD-prevention-toolbox/SCORE-Risk-Charts
